# nnUnet-based automated quantification of wrist joint synovitis volume in patients with rheumatoid arthritis: a feasibility study

**DOI:** 10.1093/radadv/umag004

**Published:** 2026-01-21

**Authors:** Bingjing Zhou, Su Wu, James Francis Griffith, Fan Xiao, Miaoru Zhang, Takeshi Fukuda, Lai-Shan Tam

**Affiliations:** Department of Imaging and Interventional Radiology, The Chinese University of Hong Kong, Hong Kong, 999077, China; CU Lab for AI in Radiology (CLAIR), The Chinese University of Hong Kong, Hong Kong, 999077, China; Department of Imaging and Interventional Radiology, The Chinese University of Hong Kong, Hong Kong, 999077, China; CU Lab for AI in Radiology (CLAIR), The Chinese University of Hong Kong, Hong Kong, 999077, China; Department of Imaging and Interventional Radiology, The Chinese University of Hong Kong, Hong Kong, 999077, China; CU Lab for AI in Radiology (CLAIR), The Chinese University of Hong Kong, Hong Kong, 999077, China; Department of Imaging and Interventional Radiology, The Chinese University of Hong Kong, Hong Kong, 999077, China; CU Lab for AI in Radiology (CLAIR), The Chinese University of Hong Kong, Hong Kong, 999077, China; Department of Imaging and Interventional Radiology, The Chinese University of Hong Kong, Hong Kong, 999077, China; CU Lab for AI in Radiology (CLAIR), The Chinese University of Hong Kong, Hong Kong, 999077, China; Department of Radiology, The Jikei University School of Medicine, Tokyo, 1638001, Japan; Department of Medicine & Therapeutics, The Chinese University of Hong Kong, Hong Kong, 999077, China

**Keywords:** rheumatoid arthritis, synovitis, wrist, MRI, segmentation, nnU-net

## Abstract

**Background:**

Synovitis is the key inflammatory feature of rheumatoid arthritis (RA). Quantitative assessment of synovitis better correlates with patient outcomes than semiquantitative assessment but it is time-consuming.

**Purpose:**

To develop and validate an automated model for segmentation and quantification of wrist synovial tissue volume on postcontrast fat-suppressed T1-weighted MRI.

**Material and Methods:**

Patients with early RA (symptoms for ≤24 months) at a single center were recruited at baseline and were followed up at year 1 and year 8. Postcontrast axial fat-suppressed T1-weighted images of the most symptomatic wrist were acquired at 3.0 T. One observer manually segmented consecutive synovitis areas on all MRI datasets. A framework, based on the convolutional neural network, nnU-Net, was trained and validated (5-fold cross-validation with image level splits) with 295 image datasets used for model training and validation. The rheumatoid arthritis MRI score was used to semiquantitatively grade synovitis. Manually segmented synovial volume by a single reader was used as the reference standard. Forty-five external image datasets from 2 different imaging centers were used to test generalizable applicability.

**Results:**

For automated synovitis segmentation, the overall Sørensen-Dice similarity coefficient (DSC) was 0.75 ± 0.11 (mean ± SD) compared to manual segmentation. Higher DSC values were found in patients with moderate (0.80 ± 0.06) and severe (0.84 ± 0.05) degrees of synovitis. The model had a similar performance with externally acquired data (DSC value: 0.70 ± 0.20). Predicted and manually segmented synovitis volume measurements showed excellent agreement (Pearson correlation: *r *= 0.975, *P *< .001).

**Conclusion:**

A fully automated model quantified wrist synovial tissue volume with good agreement to manual reference and maintained performance on external data, supporting potential use in clinical studies and prospective evaluation in practice.

Summary Statement

An automated nnU-Net model segmented and quantified wrist synovial tissue volume on postcontrast fat-suppressed T1-weighted MRI with good agreement to manual reference across internal cross-validation and 2-center external data.

Key Results

- Automated synovial volume quantification on wrist MRI yielded a Dice similarity coefficient 0.75 ± 0.11 and a small volumetric error relative to manual segmentation across 295 examinations.
- External 2-center testing (*n* = 45) achieved Dice similarity coefficient 0.70 ± 0.20 with similar volumetric agreement.
- Performance was higher in moderate and severe synovitis than in mild synovitis.

## Introduction

Synovitis is the hallmark of rheumatoid arthritis (RA).^1^^,^^2^ It is the earliest pathological feature to occur and is the main measure of inflammatory activity.^3^^,^^4^ Synovitis manifests as both synovial inflammation and synovial proliferation and can involve the joint synovium (joint synovitis) or the tenosynovium (tenosynovitis).^1^^,^^5–8^ Contrast-enhanced MRI clearly demarcates inflamed synovium from the surrounding bone and soft tissue.^9^

Many semiquantitative scoring methods have been devised for evaluating synovial severity in patients with RA.^3^^,^^10–13^ These semiquantitative methods are helpful in everyday clinical practice though they are subjective, require reader experience, cannot reflect small changes, and limit inter-study or inter-institutional comparison.^11^

Rather than a semiquantitative assessment, it would be more precise to have an actual volume measurement of synovitis. This would enable examinations to be objectively compared, particularly longitudinal examinations or examinations read by different readers. Also, objective measurement of synovial volume correlates better with clinical outcome in patients with RA than the rheumatoid arthritis MRI score (RAMRIS) semiquantitative measurement.^14^^,^^15^

Manual segmentation of synovial volume is possible by demarcating and summing synovial areas on serial MR images to obtain synovial volume (in cm^3^).^15^^,^^16^ This manual process is time-consuming, taking at least 20 minutes to complete 1 wrist.^15^^,^^17^ Machine-learning techniques have more recently been applied to evaluate aspects of rheumatoid arthritis treatment to good effect.^18^

We developed an automated method to segment and quantify synovial tissue volume, combining joint synovitis and tenosynovitis into a single “synovial tissue” class for clinical relevance. Fifty of the imaging databases (33%, 100/295) at baseline and year 1 used in the current study were also used in a previous study that developed a model to automatically quantify bone marrow edema proportion based on coronal T2WI fat-suppressed images.^19^ Internal performance was assessed using 5-fold cross-validation with image-level splits (each MRI session—baseline, 1 year, 8 years—treated as an independent sample), and generalizability was tested on datasets from 2 external centers acquired on different scanners and protocols.

## Material and methods

### Image acquisition

Patients with early RA (symptoms for < 24 months) were prospectively recruited at a single center at the Rheumatology Clinic from 2013 to 2015. This study is not a nontherapy trial and is not registered. Each participant contributed up to 3 MRI examinations at baseline, year 1, and year 8. All timepoints from an individual were assigned to the same cross-validation fold. The study was approved by the local ethics committee with written informed consent obtained from all participants. All patients fulfilled the 2010 American College of Rheumatology/European League Against Rheumatism classification criteria for RA.^20^ Patients’ data are shown in Supplementary Figure S1. Patients underwent a standardized clinical and MRI assessment at baseline and were recalled 1 and 8 years after initial presentation for identical clinical and MRI assessments. MRI of the most symptomatic wrist was performed. The MRI scanning protocol used is shown in Supplementary material 1.

### Semiquantitative evaluation of synovitis

#### RAMRIS score

Visual scoring of wrist synovitis was performed using the RAMRIS synovitis subscore.^21^ This was assessed on fat-saturated postcontrast T1-weighted axial images by 2 musculoskeletal radiologists. Both radiologists (reader 1 with 3 years and reader 2 with 6 years of experience) were blinded from clinical information. Both readers independently evaluated all the image datasets. Wrist synovitis was evaluated in 3 compartments, namely the (1) distal radioulnar joint, (2) radiocarpal joint, and (3) intercarpal and carpometacarpal joints. Each area was scored from 0 to 3 as follows: 0: no synovitis; 1: mild synovitis; 2: moderate synovitis; and 3: severe synovitis. Summing scores from all 3 areas yielded the synovitis score (maximum score of 9). A RAMRIS synovitis score of 1–3 was considered as mild synovitis, 4–6 as moderate synovitis, and 7–9 as severe synovitis. Inter- and intra-reader reliability for RAMRIS scoring was excellent (Supplementary Table S1).

### Quantitative evaluation of synovitis

#### Manual segmentation and volume calculation

Enhancing synovial areas were outlined on consecutive MRI scans by reader 1 using the open-source software ITK-SNAP and saved in NIFTI format.^22^ Reader 1 was trained and supervised by another musculoskeletal radiologist with 30 years of MRI reporting experience. Images with incomplete fat suppression or severe movement artifact were not used for manual segmentation. Manual synovial volume was calculated by the software. This segmentation and synovial volume measurement comprised the ground truth. The average manual segmentation time was calculated from the average time of manual segmentation for 30 cases.

#### Automated deep learning segmentation and volume calculation

MRI scans were converted from DICOM to NIfTI format using *dcm2niix* in Python 3.8. Segmentation was performed with nnU-Net v2, a self-configuring deep learning framework,^23^ using the 2D configuration on an NVIDIA A100 40 GB GPU. Training used 5-fold cross-validation with image-level splits, and all 5 models were ensembled during inference. A detailed description of the full nnU-Net pipeline—including configuration parameters, software/hardware specifications, postprocessing settings, and runtime analysis (median inference time 3.54 seconds per scan, interquartile range, 3.54–3.57 seconds)—is provided in Supplementary material 2.

Synovial volume was derived after segmentation. MRI scans were converted into 3-dimensional arrays of voxel intensities. A binary mask was created, labeling voxels as “1” for synovitis and “0” for no synovitis. Synovial volume was calculated by multiplying the number of voxels labeled as 1 by the voxel volume derived from image metadata. This mask indicates the region of interest (ROI) and can be visualized as an array with “True” for voxels within the ROI and “False” for voxels outside the ROI. The total number of voxels within the ROI was counted by summing the True values in the binary mask. The segmentation mask volume was calculated by multiplying the voxel count by the voxel size. The slice thickness was 3.0 mm and the interslice gap 0.3 mm; the interslice volume was not calculated. For automated segmentation, the average time is calculated for the time interval (input and output) divided by the number of cases input into the model.

## Validation of model with external data

Wrist MRI data were acquired from 2 other institutions, 1 of which was a local hospital and the other a university hospital in another country. These external MRI studies had also been performed to assess synovitis in patients with RA and included postcontrast T1-weighted fat-suppressed axial wrist images. The MRI systems and the imaging parameters used in each of the external institutions were different from the parameters used in the base institution. External data underwent the same manual and automated segmentation and calculation of synovial volume as had been done for internal data. MRI scanning protocols for external sites are detailed in Supplementary Material 1.

### Statistical analysis

Mean and SD were used for normally distributed data. Median with interquartile range was used for skewed data. Inter- and intra-class correlation analysis were used to test RAMRIS score reliability. The paired *t*-test was used to compare manual and automated synovial volume measurements. Pearson’s correlation coefficient was used to evaluate the correlation between manual and automated synovial volumes. Bland-Altman plots were drawn to define agreement between manual and automated methods. Two 1-sided tests were used for equivalence analysis. A *P* value < .05 was considered statistically significant.

### Code availability

All experiments were conducted with the open-source nnU-Net v2 framework.^23^ The full implementation, installation instructions, and training/inference utilities are publicly available on GitHub: https://github.com/MIC-DKFZ/nnUNet. The exact configuration and commands used in this study are documented in the Supplementary Material 2 and provided as reproducible scripts with the manuscript.

## Results

### Image acquisition

**Figure 1:**
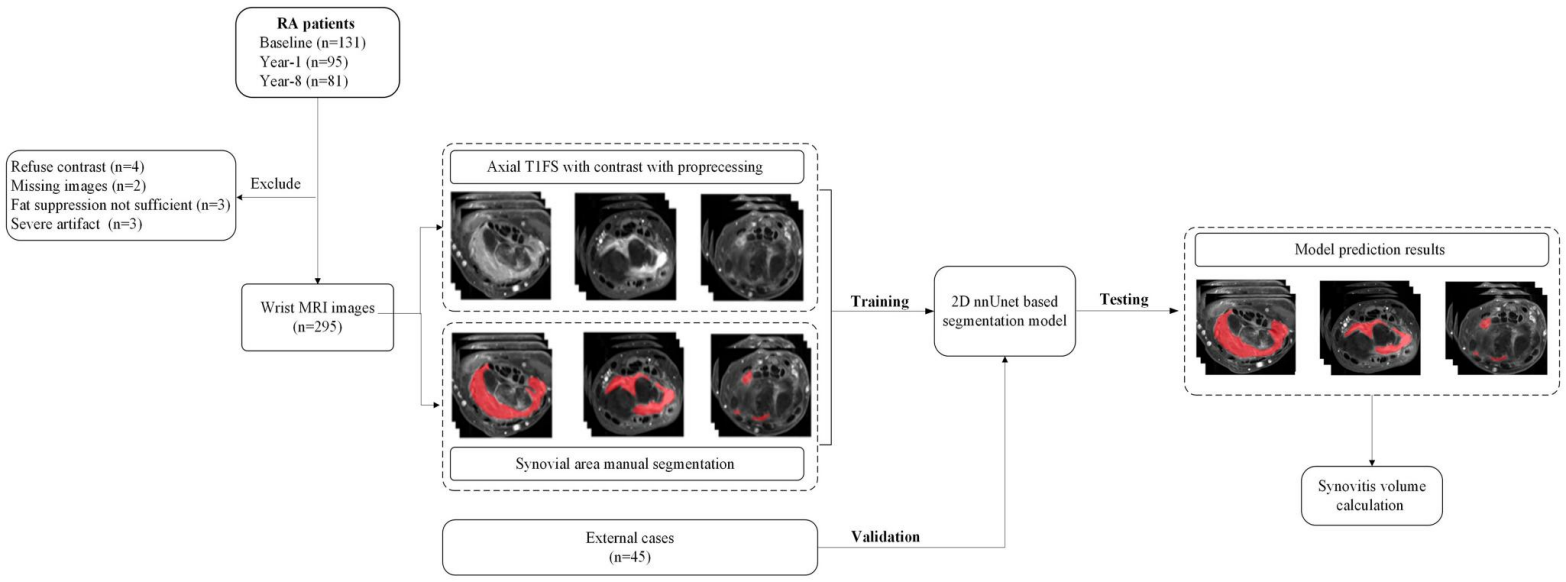
Study flowchart. A total of 295 datasets were used for model training and testing. Forty-five external cases were acquired to test external validation.

One hundred and thirty-one, 95 (73%), and 81 (62%) patients completed the baseline, year 1, and year 8 follow-up assessments respectively. In total, 307 MRI examinations were acquired. Twelve (4%) of the 307 image datasets were excluded because of inadequate fat suppression or severe motion artefact, leaving 295 image datasets for model training (Figure 1). Average manual segmentation time for 1 case was 9.9 ± 4.8 (SD) minutes, whereas average time for model segmentation was 3.54 seconds per case (interquartile range: 3.54–3.57 seconds). Patient details are summarized in Table 1.

**Table 1:** Patient demographics at baseline, year 1, and year 8.

	Baseline	Year 1	Year 8
Patient characteristic	(*n* = 131)	(*n* = 95)	(*n* = 81)
Age (years)^a^	53.6 ± 12.6	56.2 ± 12.3	61.0 ± 12.6
Sex (F/M)	100/31	76/19	67/14
Right wrist^b^	78	59	50

F = female, M = male.

a Mean ± SD.

b The most symptomatic wrist was examined at baseline and the same wrist at year 1 and year 8.

### Segmentation results

**Figure 2:**
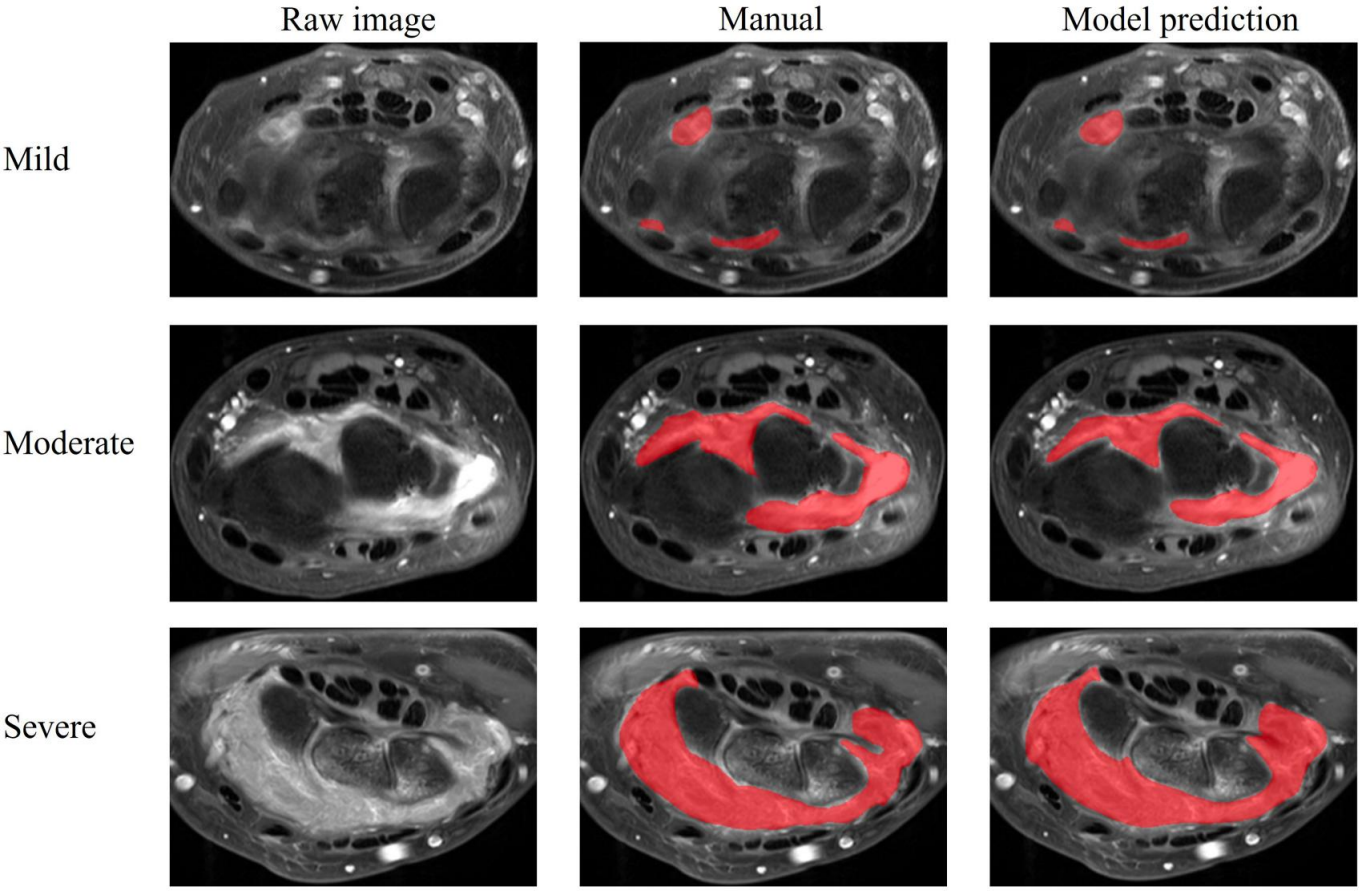
Examples of manual segmentation in patients with mild, moderate, and severe degrees of synovitis. The red area represents the synovitis area.

**Figure 3:**
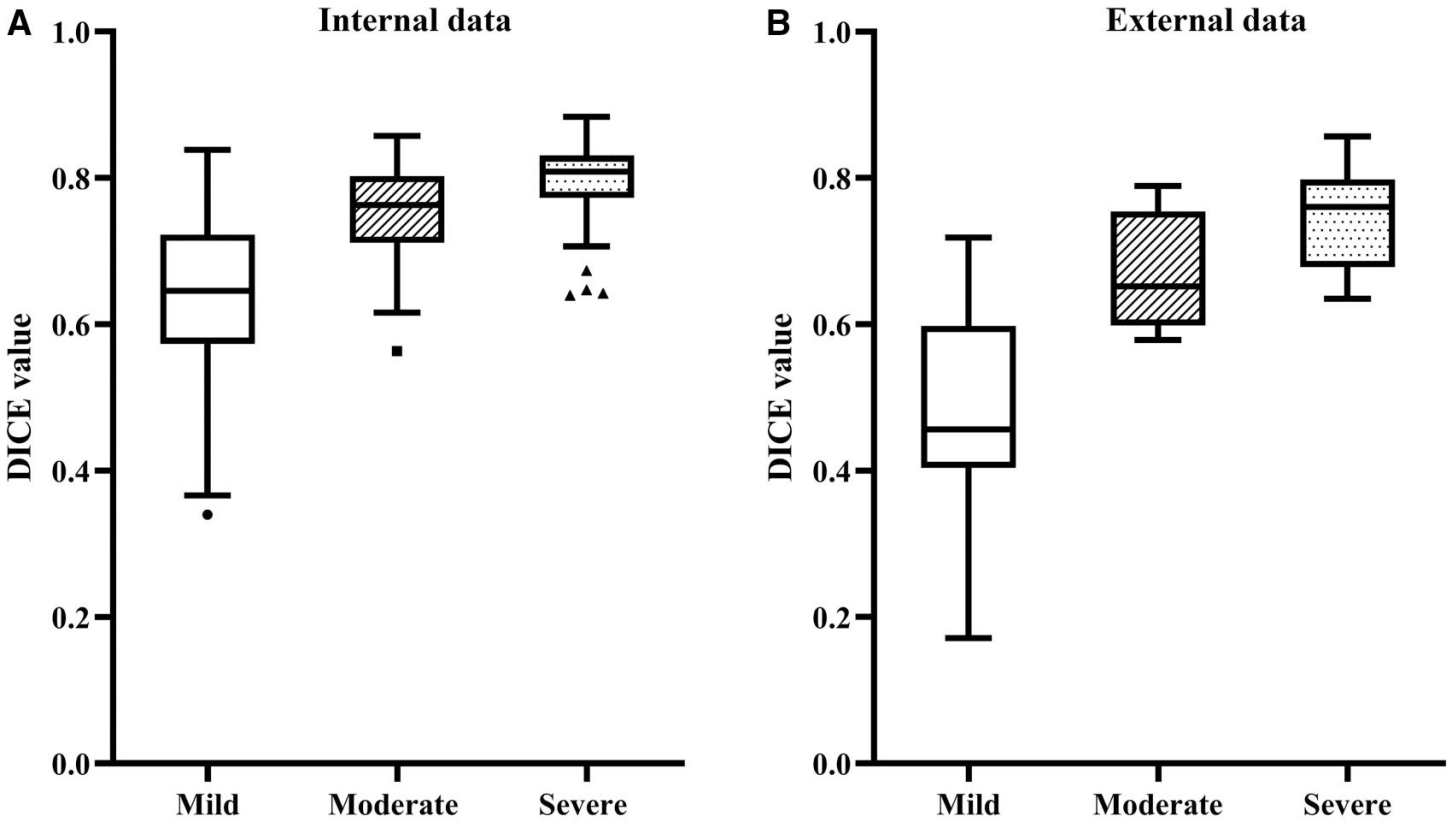
Box-and-whisker plot of DSC values in different synovitis severity groups for internal (A) and external data (B). The dot represents the outliers of each group; the line represents the median DSC value for each group. Moderate and severe synovitis-severity showed higher DSC values than mild-severity synovitis. DSC = Dice similarity coefficient.

Figure 2 shows examples of mild, moderate, and severe synovitis with the corresponding manual labels and automated nn-UNet model segmentation results. Overall Sørensen-Dice similarity coefficient (DSC) value was 0.75 for all image datasets comparing manual segmentation and model segmentation results. The model showed better segmentation performance for moderate and severe synovitis, based on RAMRIS score, than mild synovitis (Table 2). Mild synovitis had a mean DSC score of 0.68, moderate synovitis had a score of 0.80, and severe synovitis had a score of 0.84 (*P *< .001) (Figure 3) (Table 2).

**Table 2:** Dice similarity coefficient (DSC) value for different synovitis groups and overall for synovial volume prediction model.

	RAMRIS synovitis score			
	Mild	Moderate	Severe	*P* value
Number of patients	139	75	81	
Manual segmentation volume (cm^3^)	1.76 ± 1.44	5.20 ± 3.19	10.79 ± 4.42	<.001
Model calculation volume (cm^3^)	1.61 ± 1.39	4.99 ± 3.28	9.96 ± 4.10	<.001
Mean DSC	0.68 ± 0.11	0.80 ± 0.06	0.84 ± 0.05	<.001
Overall DSC	0.75 ± 0.11			

Data are shown as mean ± SD.

RAMRIS = rheumatoid arthritis MRI score.

### Synovial volume calculation

**Figure 4:**
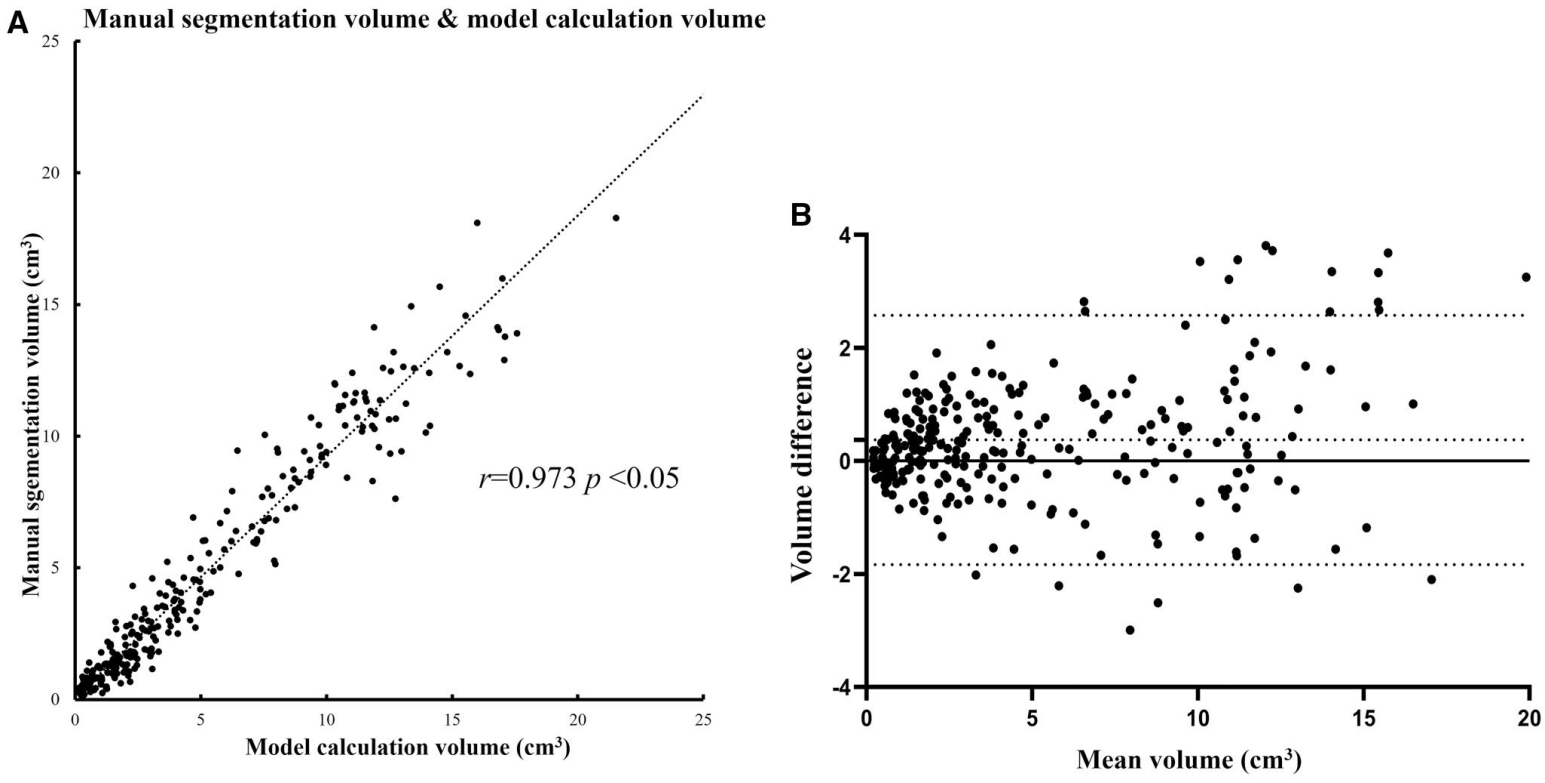
(A) Scatter plot of model prediction volume and manual segmentation volume. Each dot represents a single patient. Pearson correlation *r* = 0.973, *P* < .001. Dashed black line represents linear regression fit. (B) Bland-Altman plot of manual and automated synovial volume calculations. The Y axis represents the difference between the 2 methods and the X axis represents the mean of both methods. The dotted line in the middle represents the overall bias between the 2 methods. When this line is close to 0, the agreement between both methods is good. The bias between manual and automated volume calculation is 0.37 (95% CI, -1.83 to 2.58).

Synovial volume for manual segmentation was 5.3 ± 4.8 cm^3^. Automated model synovial volume was 4.9 ± 4.6 cm^3^ (paired *t*-test, *P *< .001). Pearson’s correlation analysis showed excellent correlation between manual and automated synovial volume measurements (*r *= 0.973, *P *< .001) (Figure 4a). The Bland-Altman plots showed good agreement between manual and automated synovial volume measurements; the bias was 0.37 (95% CI [–1.83 to 2.58]) (Figure 4b). Two 1-sided tests showed that the automated model was equivalent to the manual model (*P *= .0197 for inferiority test and *P* < .001 for superiority test).

### External validation

**Figure 5:**
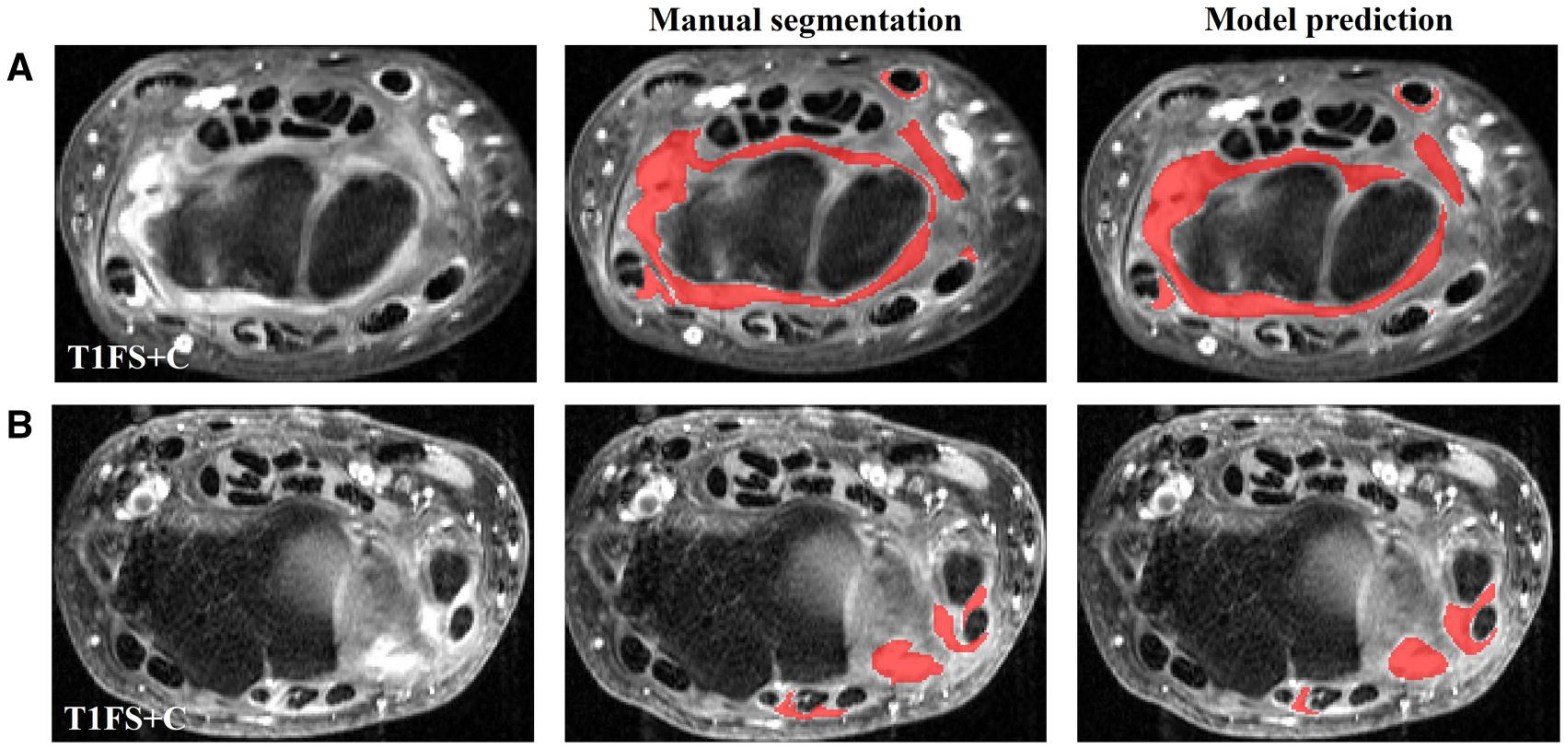
(A) A 53-year-old female patient with RA from another hospital. The scanning protocol was different from that used for internal data but the model showed similar segmentation results compared with manual segmentation with a DSC value of 0.72. (B) A 56-year-old female patient with RA from another hospital. This patient has mild synovitis with a DSC value of 0.42. DSC = Dice similarity coefficient; RA = rheumatoid arthritis.

Patient details of the external datasets are shown in Table 3. The model had a similar prediction performance for external data as internal data with a mean synovial volume DSC value of 0.70 (Figure 5). Severe synovitis had a higher DSC value than moderate and mild synovitis. Correlation between synovial volumes of external data predicted by the nnU-Net model and manual segmentation was also excellent (*r *= 0.979, *P *< .001).

**Table 3:** Patient characteristics of external validation datasets.

Patient characteristic	(*n* = 45)
Age	55.0 ± 11.8
Sex (F/M)	33/12
Right side	30
Manual segmentation volume (cm^3^)	8.18 ± 8.15
Model calculation volume (cm^3^)	7.87 ± 7.03
DSC value	
Mild synovitis (*n* = 17)	0.54 ± 0.17
Moderate synovitis (*n* = 8)	0.69 ± 0.07
Severe synovitis (*n* = 20)	0.75 ± 0.09
Overall	0.70 ± 0.20

DSC = Dice similarity coefficient, F = female, M = male.

### RAMRIS score and synovium volume

RAMRIS score showed good correlation with synovium volume (*r* = 0.80, *P * < .001) for both internal and external data. For RAMRIS patients with mild, moderate, and severe degrees of synovitis, average synovium volume was 1.8 cm^3^, 5.2 cm^3^, and 10.8 cm^3^, respectively.

## Discussion

Our study developed a method of automated measurement of synovial volume. Tenosynovitis was not separated from synovitis in either the manual or automated segmentation processes as: (1) total synovial volume better reflects inflammation and response to treatment than synovitis or tenosynovitis in isolation; and (2) separating tenosynovitis from synovitis lowered, rather than improved, prediction performance, with derived DSC values varying from 0.40 to 0.60.

More severe synovial inflammation is related to more severe structural damage progression and worse clinical outcome in patients with RA.^10^^,^^14^^,^^24^ Synovial inflammation or synovitis can be readily seen on MRI. In everyday clinical practice, synovitis is subjectively classified as mild, moderate, or severe. In research studies, synovitis is semiquantitatively graded using the RAMRIS system, along with an accompanying reference atlas.^21^ Manual quantitative measurement of wrist synovial volume is feasible but it takes at least 20 minutes to complete each wrist and is therefore not practical in everyday clinical settings or in large data research studies.^25^

The model developed can reliably measure synovial volume (in cm^3^) in less than 1 minute. We believe that this model will become widely used in clinical trials initially and later in everyday clinical practice as a measure to objectively assess the level of synovial inflammation in individual patients, helping to enable ready comparison across different MRI studies and providing an objective method to serially track synovial volume as a response to treatment.

The model showed better performance for patients with moderate and severe synovitis than for patients with mild synovitis. Compared to moderate or severe synovitis cases, mild synovitis tends to have more ill-defined margins and less significant enhancement. In the calculation of the DSC value, only the overlapping areas between manual segmentation and automated model prediction are considered as correct. Slight differences in the synovitis margin will be considered incorrect and will lead to a decrease in DSC value.

To help demonstrate the potential for general use of this program in everyday clinical practice and clinical trials, the model was tested against 2 sets of external data, which were acquired on different MRI systems with slightly different imaging protocols. Despite these differences, the model fared well in assessing synovial volume compared to manual segmentation. This indicates that the model has the potential for wide usage. Similar to internal data, the correlation between manual and automated synovial volume measurements was better for external data with moderate or severe synovitis rather than mild synovitis.

Two prior studies have automatically quantified synovial and/or tenosynovial severity in the wrist joint of patients with RA.^26^^,^^27^ One study^26^ devised a model to automatically quantify tenosynovitis severity. High correlation (*r* = 0.90, *P *< .001) between visual and automated assessment of tenosynovitis severity was found but synovitis and vessels led to the false detection of tenosynovitis in automatic measurements. A more recent single-center study of only 12 patients with RA used a different method to automatically grade synovitis severity.^27^ Instead of using morphological features to define synovitis, this study used the signal intensity curve pattern for each pixel on dynamic contrast-enhanced MRI to differentiate highly perfused tissues. The model was not compared with manual-segmented volumes though it did show excellent correlation with RAMRIS score.^27^ The model was also not externally validated and did not provide a measure of synovial volume (in cm^3^) per se.^27^ Other synovitis segmentation studies have focused on the hand and knee joints.^27–29^ The hands and knees are easier to segment than the more clinically relevant wrist joint as the wrist joint comprises many small bones that makes segmentation more challenging.^30^^,^^31^

Our study has some limitations. First, the number of image datasets (*n* = 295) was modest. To obtain a more stable and generalizable estimate of model performance on unseen data, we adopted an image level 5-fold cross-validation procedure, allowing each dataset to contribute to both training and validation across different folds. Although cross-validation does not increase dataset size, it reduces performance variance and mitigates the risk of bias because of arbitrary data splits. Second, in this study, we lack a patient level validation and we chose image level validation rather than patient level validation, which may have a risk of data leakage. We chose to use image level validation as (1) the sample size is small and would have been smaller had a patient level validation being used and (2) the degree of synovitis can vary considerably with treatment from 1 examination to the next which can minimize the effects of using repeated examinations and (3) we compared the model performance for patients with and without follow-up (Supplementary material Table S2), as patients with follow-up have a theoretical risk of image leakage, but it did not show a significant difference in performance, which indicates that image level validation did not overestimate the model performance. Third, the model addressed wrist synovitis as the wrist is the most affected joint in patients with RA and is the best marker of systemic inflammatory activity in patients with RA.^1^ Similar models could be developed to address synovitis in other joints such as the small joints of the fingers, the hip, or the knee. Fourth, in this study, 1 observer measured all of the manual segmentation, potentially compromising model performance.

In conclusion, we present a reliable method of automatically segmenting and quantifying wrist joint synovial volume in patients with RA based on T1-weighted fat-suppressed postcontrast MRI scans. Automated quantification of synovial volume can be completed in less than a minute, has a high correlation with both manual segmentation and semiquantitative RAMRIS scores, and has good performance when tested on external data.

## Supplementary Material

umag004_Supplementary_Data

## Data Availability

The data underlying this article will be shared on reasonable request to the corresponding author.
