# Supplementary material for: nnUnet-based automated quantification of wrist joint synovitis volume in patients with rheumatoid arthritis: a feasibility study
Source: Radiol Adv. 2026 Jan 21;3(2):umag004. doi: 10.1093/radadv/umag004 (PMC12975715; doi:10.1093/radadv/umag004)
Supplement: umag004_Supplementary_Data [file umag004_supplementary_data.zip › Supplement_Data.zip!/Supplementary material.docx]

**nnUnet-based automated quantification of wrist MRI joint synovitis volume in RA patients: a feasibility study**

^1^Bingjing Zhou, BEng

^1^Su Wu, MMed

^1^James Francis Griffith, MB, BCh, BAO, MD, MRCP, FRCR, FHKCR, FHKAM

^1^Fan Xiao, PhD

^1^Miaoru Zhang, MMed

^2^Takeshi Fukuda, MD, PhD

^3^Lai-Shan Tam, MRCP, MD

**^1^Department of Imaging and Interventional Radiology, The Chinese University of Hong Kong, Hong Kong, China** email: [radiology@med.cuhk.edu.hk](mailto:radiology@med.cuhk.edu.hk)

**^1^CU Lab for AI in Radiology (CLAIR), The Chinese University of Hong Kong, Hong Kong SAR, China**

Address: Rm 27023, LG/F, Prince of Wales Hospital, N.T., Hong Kong

^2^**Department of Radiology, The Jikei University School of Medicine**

email: takenet616@gmail.com

Address: 3-25-8, Nishi-Shimbashi, Minato-Ku, Tokyo, Japan.

**^3^Department of Medicine & Therapeutics, The Chinese University of Hong Kong, Hong Kong, China**

email: medt@cuhk.edu.hk

Address: 9/F, Lui Che Woo Clinical Sciences Building, Prince of Wales Hospital, Shatin, N.T., Hong Kong

**Correspondence to:** James Francis Griffith. Department of Imaging and Interventional Radiology, The Chinese University of Hong Kong, Hong Kong, China.

Email: [griffith@cuhk.edu.hk](mailto:griffith@cuhk.edu.hk)

Tel: 3505 1167

**Supplementary figure S1:**

Flowchart of patient enrolment at baseline, year-1 and year-8. Sixty-four patients completed the baseline, year-1 and year-8 assessments. 17 patients did not undergo year-1 follow-up. #Excluded at year-1 due to diagnosis of systemic lupus erythematosus (n=3) and peripheral spondylarthritis (n=1) * Contrast enhanced MRI was contradicted because of implant (n=3), claustrophobia (n=1), malignant tumour (n=4), kidney failure (n=1), and age more than 90 years (n=1). There was no difference in clinical, serological, radiographic or MRI parameters at baseline between the 50 patients who did not complete and the 81 patients who did complete the year-8 follow up (*p* > 0.05 for all).

**Supplementary material 1:**

MRI scanning protocol for internal images:

Wrists were scanned in a Superman position on a 3.0-T system (Phillips Integra, Best, Netherlands) using a dedicated wrist coil and a scan plane extending from the distal radio-ulnar joints to the metacarpal bases. The following sequences were obtained: fat-saturated T1-weighted axial (TR 692ms; TE 12ms; echo spacing 12ms; flip angle 90°; FOV 80×80mm; slice thickness 3mm; NEX 2); fat-saturated T2-weighted coronal (TR 3121ms; TE 70ms; echo spacing 10ms; flip angle 90°; FOV 80×80mm; slice thickness 1.5mm; NEX 1); T1-weighted coronal (TR 547ms; TE 12ms; echo spacing 12ms; flip angle 90°; FOV 80×80mm; slice thickness 1.5mm; NEX 1); fat-saturated post-contrast T1-weighted axial (TR 692ms; TE 12ms; echo spacing 12ms; flip angle 90°; FOV 80×80mm; slice thickness 3mm; NEX 2).

| Scanning protocol | External hospital 1 | External hospital 2 |
| --- | --- | --- |
| Manufacture | Simens | Simens |
| Field strength | 3.0T | 3.0T |
| TR (ms) | 4150 | 460 |
| TE (ms) | 66 | 13 |
| Flip angle | 90° | 90° |
| Bandwidth (Hz per pixel) | 220 | 185 |
| Matrix size (mm) | 384×269 | 448×358 |
| FOV (mm*mm) | 100×100 | 100×100 |
| Slice thickness (mm) | 2.5 | 3 |
| Slice gap (mm) | 0.3 | 0.3 |

MRI scanning protocol for external images:

**Supplementary table S1**:

Inter- and intra- class correlation coefficient results of synovitis score for reader 1 and reader 2 (n=145)

|  | Synovitis score |
| --- | --- |
| Inter-class correlation | 0.833 |
| Intra-class correlation |  |
| Reader 1 | 0.894 |
| Reader 2 | 0.909 |

**Supplementary table S2**:

Pearson’s correlation coefficient results for patients who participate and who did not participate in the follow-up study.

|  | Patient without follow-up (n=53) | Patient with follow-up (n=78) |
| --- | --- | --- |
| Manual segmentation volume(cm^3^) | 8.0±5.6 | 7.0±5.1 |
| Model calculation volume(cm^3^) | 7.5±4.4 | 6.9±5.4 |
| DICE value | 0.81±0.05 | 0.83±0.05 |
| Pearson’s correlation coefficient | 0.971 | 0.958 |

**Supplementary material 2:Deep Learning Pipeline Specification**

**1. Model Configuration (nnU-Net v2 Auto-Configuration)**

All segmentation models were trained using the **2D nnU-Net v2 framework**, which performs full self-configuration based on dataset properties as described by Isensee et al. (23).

The auto-generated settings defined:

- **Preprocessing:** DefaultPreprocessor
- **Intensity normalization:** Per-channel Z-score
- **Network:** PlainConvUNet (standard nnU-Net encoder–decoder)
- **Depth, number of pooling levels, convolutional blocks, feature channels:** Determined automatically according to nnU-Net’s planning algorithm
- **Patch size, batch size, resampling strategy:** Automatically selected from dataset analysis
- **Loss function:** Dice-based loss with batch-wise Dice enabled
- **No manual overrides** were applied to any architectural or preprocessing parameters.

**2. Dataset-Specific Planning**

The experiment was initialized using nnU-Net’s automated ExperimentPlanner, which extracted dataset statistics (voxel spacing, median image size, and foreground intensity distribution) and produced a dataset-specific plan.

This plan defined:

- Image resampling behavior
- Network resolution hierarchy
- Normalization strategy
- Augmentation policy
- Memory-constrained patch sizing
- Data loader configuration

All settings followed nnU-Net v2 defaults; no custom changes were introduced.

**3. Data Augmentation (nnU-Net Defaults)**

Training augmentation used nnU-Net’s standardized, validated pipeline:

- **Spatial:** rotations, scaling, mirroring, elastic transforms
- **Intensity:** brightness/contrast shifts, gamma augmentation
- **Noise/blur:** Gaussian noise and smoothing
- **Sampling:** random cropping from planned patch size

All augmentations were automatically tuned by nnU-Net according to dataset characteristics.

**4. Training Procedure**

- **Cross-validation:** 5-fold, image-level split (as described in Methods)
- **Optimizer:** Stochastic Gradient Descent with Nesterov momentum (nnU-Net default)
- **Learning rate schedule:** nnU-Net’s polynomial decay
- **Epochs:** Determined automatically according to nnU-Net policies
- **Ensembling:** Predictions from all 5 folds combined for final inference
- **Seed:** nnU-Net v2 default random seed behavior

All hyperparameters followed nnU-Net’s standardized recipe, ensuring reproducibility.

**5. Inference and Postprocessing**

Inference incorporated:

- **Sliding-window tiling** (automatic nnU-Net setting)
- **Test-time augmentation (TTA)**
- **Five-fold ensemble averaging**
- **Automated postprocessing** selected based on cross-validation behavior

No manual tuning was performed.

**6. Software and Hardware Environment**

- Framework: **nnU-Net v2**
- Deep learning backend: **PyTorch**
- Python version: **3.8**
- Operating system: **Linux-based HPC cluster**
- GPU: **NVIDIA Tesla A100 (40 GB)**
